# An *n*-Type Ionic Thermoelectric Device Enabled by Synergistic Interactions Between Electrodes and PVA Hydrogel

**DOI:** 10.3390/ma19102029

**Published:** 2026-05-13

**Authors:** Changsheng Ye, Xin Shan

**Affiliations:** Key Laboratory of Advanced Civil Engineering Materials (Ministry of Education), School of Materials Science and Engineering, Tongji University, Shanghai 201804, China; 2331565@tongji.edu.cn

**Keywords:** *n*-type ionic thermoelectric, thermodiffusion, PVA hydrogel, electrodes

## Abstract

Ionic thermoelectric (i-TE) materials have attracted increasing attention for low-grade heat harvesting owing to their high thermovoltage output under small temperature gradients. However, the development of *n*-type i-TE materials remains challenging. Electrode-enabled polarity regulation provides a promising alternative to material-design strategies for developing *n*-type i-TE devices. In this work, a poly(vinyl alcohol) (PVA)-based ionic hydrogel was prepared with dimethyl sulfoxide (DMSO) and potassium chloride (KCl) through a freeze–thaw process, and its thermoelectric behavior was regulated by electrodes. While the i-TE hydrogel device with typical Cu electrodes exhibited *p*-type behavior, replacing the electrodes with graphite paper (GP) electrodes converted the device response from *p*-type to *n*-type. Morphological and spectroscopic analyses suggest that the GP surface selectively adsorbed K^+^ ions through cation–π interactions, suppressing cation thermodiffusion and enabling Cl^−^-dominated ion migration under a temperature gradient. As a result, the PVA-GP device achieved a maximum *S_i_* of −4.36 ± 0.26 mV K^−1^. In addition, the device exhibited favorable thermoelectric output, with a maximum *PF_i_* of 57.668 μW m^−1^ K^−2^, a room-temperature *ZT* of 0.0864, and a peak transient power density of 2.33 mW m^−2^ during short-time discharge. Owing to the large interfacial area of the GP electrodes, the device could also function as an ionic thermoelectric supercapacitor with appreciable energy-storage capability. This work demonstrates an effective electrode-engineering strategy for constructing *n*-type i-TE devices and provides a feasible route for simultaneous low-grade heat harvesting and transient energy storage.

## 1. Introduction

Large amounts of low-grade heat are continuously dissipated from chemical plants, solar thermal systems, and daily human activities. Efficient recovery and utilization of this energy are important for improving energy efficiency and promoting sustainable development [1,2]. With the rapid advancement of the Internet of Things and wearable electronics, thermoelectric (TE) technology, which enables direct conversion of heat into electricity, has gradually emerged as a promising alternative energy solution [3,4]. Conventional electronic thermoelectric materials are constrained by their relatively low Seebeck coefficients (*S_i_*), which limits their efficient operation under small temperature gradients [5,6]. In contrast, ionic thermoelectric (i-TE) technologies driven by ionic thermodiffusion, also known as the Soret effect, or by redox reactions (thermogalvanic effect), can generate thermovoltages several orders of magnitude higher than those of electronic thermoelectric devices because ionic enthalpy is far greater than electronic enthalpy [7,8,9,10]. As a result, i-TE technologies have shown great potential for the efficient and low-cost harvesting of low-grade thermal energy. Owing to their excellent flexibility, processability, and thermal stability, hydrogels and ionogels composed of a solid polymer matrix that provides structural support, together with inorganic salts or ionic liquids that serve as the dispersion medium and ion source, have become the mainstream material choices for fabricating i-TE devices [11,12,13].

To date, i-TE devices based on *p*-type i-TE materials have been extensively investigated. In these materials, cations migrate faster than anions under a temperature gradient, thereby generating a positive thermovoltage [14,15,16,17]. For example, Shi et al. developed a highly tough i-TE device based on a P(N-acryloylsemicarbazide-co-acrylic acid) (PNA) supramolecular hydrogel. Through the synergistic coupling of ionic thermodiffusion and thermoelectrochemical reactions, the thermoelectric output of the device was significantly enhanced, delivering an *S_i_* as high as 40.9 mV K^−1^ [18]. However, it should be noted that the thermoelectric performance of a single i-TE device is generally still insufficient for practical applications [11,19,20,21]. Therefore, multiple *p*-type and *n*-type i-TE elements are typically connected in series electrically and in parallel thermally to achieve sufficient energy output [22,23,24]. Nevertheless, the development of *n*-type i-TE materials with negative thermovoltage output has remained less developed [25].

In recent years, various strategies have been proposed to fabricate *n*-type i-TE materials, including regulating polymer–ion interactions, incorporating inorganic fillers, and introducing ionic dopants [17,26,27,28]. For instance, Le et al. used poly(vinylidene fluoride-co-hexafluoropropylene) (PVDF-HFP) as the matrix, 1-butyl-3-methylimidazolium tetrafluoroborate (BmimBF_4_) as the ion donor, and silver trifluoromethanesulfonate (AgOTf) as the dopant, achieving a maximum *S_i_* of −26.4 mV K^−1^ [29]. Yu et al. prepared a PVA-based *n*-type i-TE hydrogel by simultaneously introducing CuCl_2_ and PMNT. By utilizing the coordination interaction between Cu^2+^ and the hydroxyl groups of PVA, together with the hydrophobic interaction between PVA and PMNT, they achieved a highly negative *S_i_* of up to −38.6 mV K^−1^ [30].

At present, increasing attention has been directed toward regulating thermovoltage through interactions at the electrode/electrolyte interface [31,32]. Previous studies have shown that different electrodes can markedly affect the thermovoltage output of i-TE devices. For example, Horike et al. reported an ionogel based on EmimCl and PVA, and found that a stable thermovoltage could be generated when Ag electrodes were used, whereas only a transient and weak voltage response was observed with Au electrodes. This behavior was attributed to thermally induced interfacial polarization [33]. Mardi et al. employed an EmimTFSI/PVDF-HFP ionogel as the electrolyte and, based on the anion-repelling effect of PSS, namely the Donnan effect, tuned the PSS content in PEDOT-PSS electrodes to achieve a maximum *S_i_* of −4.6 mV K^−1^ [28]. More importantly, Chi et al. demonstrated that a composite composed of PVDF-HFP, PC, and NaTFSI could exhibit two opposite i-TE behaviors, namely *p*-type and *n*-type, simply by replacing the external electrodes. These findings clearly indicate that electrodes not only influence the magnitude of i-TE performance, but can even determine the p/n polarity of the device [34]. This observation is particularly noteworthy. Compared with developing new *n*-type matrix materials, electrode-based regulation may provide a new and promising route for the low-cost, large-scale construction of high-performance *n*-type i-TE materials.

Herein, we report an i-TE hydrogel prepared from PVA, dimethyl sulfoxide (DMSO), and potassium chloride (KCl) via a freeze–thaw cycling method. It was found that the device exhibited *p*-type behavior when conventional metallic copper sheets were used as the electrodes. When the electrodes were replaced with GP, the selective adsorption of K^+^ by the GP electrodes restrained the thermodiffusion of K^+^, thereby converting the device into an *n*-type system. The i-TE device assembled with GP electrodes (PVA-GP) achieved a maximum *S_i_* of −4.36 ± 0.26 mV K^−1^ and delivered a maximum power density of 2.33 mW m^−2^ at a temperature difference of 10 K. In addition, owing to the large specific surface area of the GP electrodes, PVA-GP can also function as an ionic thermoelectric supercapacitor with appreciable energy-storage capability. The PVA hydrogel can be readily prepared, and an *n*-type i-TE device can be realized simply by employing inexpensive and readily available GP electrodes. This work therefore provides a promising and feasible strategy for the low-cost, large-scale construction of high-performance *n*-type i-TE systems for intermittent and short-term energy harvesting applications.

## 2. Materials and Methods

### 2.1. Experimental Materials

Polyvinyl alcohol type 1799 (degree of alcoholysis: 98–99%, average molecular weight: 74,800–76,000 g mol^−1^) was purchased from Aladdin Co., Ltd. (Shanghai, China). Dimethyl sulfoxide (AR) and potassium chloride (GR) were purchased from Sinopharm Chemical Reagent Co., Ltd. (Shanghai, China). All reagents were used as received without further purification. Graphite paper was supplied by Kunshan Research Metal Technology Co., Ltd. (Kunshan, China).

### 2.2. Preparation of PVA-Based Hydrogels

PVA-based hydrogels with different KCl concentrations (0.1, 0.3, 0.5 and 0.8 M) were prepared using the same procedure. Briefly, deionized water and DMSO were mixed at a fixed mass ratio of 7:3 to obtain 20 mL of solvent. KCl was then added according to the target concentration, followed by stirring for 15 min. Subsequently, 2.857 g of PVA was added, and the mixture was stirred at 90 °C for 3 h and degassed at 90 °C for 30 min. The solution was poured into a polytetrafluoroethylene mold and subjected to one freeze–thaw cycle, consisting of freezing at −20 °C for 20 h and thawing at 25 °C for 3 h. Unless otherwise specified, all hydrogels used for thermoelectric measurements were prepared into 30 × 30 × 3 mm samples.

PVA-DMSO hydrogels with different DMSO contents were also prepared for comparison. The samples were denoted as PVA-DMSO_0_, PVA-DMSO_30_, and PVA-DMSO_50_, where the subscript indicates the mass fraction of DMSO in the water/DMSO binary solvent.

### 2.3. Characterization and Measurements

The surface microstructure and pore structure of the hydrogels were characterized using field-emission scanning electron microscopy (FE-SEM, ZEISS GeminiSEM 300, Oberkochen, Germany), and elemental analysis was further conducted by energy-dispersive spectroscopy (EDS). The chemical functional groups of the hydrogels were analyzed by Fourier-transform infrared spectroscopy (FT-IR, Thermo Fisher Scientific Nicolet iS20, Waltham, MA, USA). The elemental composition and relative contents on the surfaces of both the hydrogels and the electrodes were quantitatively analyzed using X-ray photoelectron spectroscopy (XPS, Thermo Scientific K-Alpha, Waltham, MA, USA). The Raman spectra of the electrode surfaces were collected using a Horiba LabRAM Odyssey spectrometer (Palaiseau, France). The crystal structure and phase composition of the hydrogels and electrodes were characterized by X-ray diffraction (XRD, Rigaku Ultima IV, Akishima, Japan). The thermal conductivity of the hydrogels was measured using a thermal conductivity analyzer (XIATECH TC3000E, Xi’an, China). The electrochemical performance of the hydrogels was systematically evaluated with an electrochemical workstation (CHI760E, Shanghai Chenhua Instrument Co., Ltd., Shanghai, China).

### 2.4. Thermoelectric Performance Characterization

**Figure 1:**
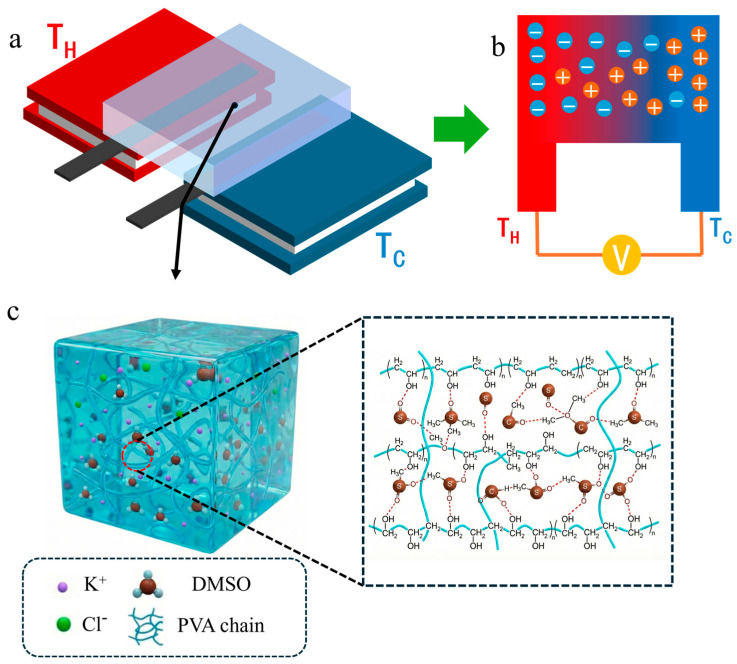
(**a**) Ionic thermoelectric testing device and schematic diagram of its principle; (**b**) mechanism of the directional diffusion of cations and anions within the hydrogel under a temperature gradient; (**c**) schematic illustration of the three-dimensional polymer network structure of the PVA-DMSO hydrogel.

A custom-built setup for thermoelectric performance measurements is shown in Figure 1a. All measurements in this study were performed on hydrogels with dimensions of 30 × 30 × 3 mm at room temperature (298 K, 60% relative humidity). To comprehensively evaluate the thermoelectric performance of the hydrogel, a temperature gradient was applied by placing the heat sources in parallel at the left and right sides of the hydrogel. This horizontal configuration reduces convective heat loss between the hot and cold sides and minimizes gravitational effects on ion distribution or flow, thereby enabling the measured results to more accurately reflect the intrinsic i-TE performance of the material. The Peltier devices, purchased from Shenzhen Tecooler Technology Co., Ltd. (Shenzhen, China), were arranged horizontally and their temperatures were controlled by a temperature control module (TEC207, Sensefuture (Shenzhen) Technology Co., Ltd., Shenzhen, China). Two strip-shaped GP electrodes, each with a width of approximately 10 mm, were first ultrasonically cleaned in ethanol for 15 min prior to the experiments to remove surface impurities. The electrodes were placed in parallel on the Peltier devices with an interelectrode distance of 5 mm. The PVA hydrogel was then positioned on top of the electrodes, and the thermovoltage and temperature were recorded using a high-precision data logger (DAQ6510, Tektronix, Beaverton, OR, USA). In this study, the thermovoltage (Δ*V*) is defined as follows:

(1) $$\Delta V=V_{C}-V_{H}=∆V$$

where *V_C_* and *V_H_* are the potentials at the cold and hot sides, respectively.

The Seebeck coefficient (*S_i_*) is defined as:

(2) $$S_{i}=\frac{\Delta V}{T_{H}-T_{C}}$$

where *T_H_* and *T_C_* are the temperatures at the hot and cold sides, respectively.

The temperature difference across the two ends of the hydrogel was controlled by the temperature control module, and the corresponding thermovoltage under each temperature difference was recorded. The *S_i_* was obtained from the slope of the linear fitting of Δ*V* versus Δ*T*, where Δ*T* was varied from 2 to 10 K. At each Δ*T*, the voltage was recorded after reaching a stable value.

The ionic conductivity of the hydrogel was measured by electrochemical impedance spectroscopy (EIS). The hydrogel sample was sandwiched between two stainless-steel electrodes, each with an effective area of 10 × 30 mm, and tested using an electrochemical workstation. The frequency range was set from 100 kHz to 1 Hz. The ionic conductivity (*σ*) was calculated as follows:

(3) $$\sigma =\frac{L}{R\cdot S}$$

where *L* is the distance between the two electrodes, *R* is the bulk resistance, and *S* is the effective contact area between the electrodes and the hydrogel. The *σ* of the hydrogel was determined by averaging the values obtained from multiple independent measurements.

To evaluate the output performance of the device, linear sweep voltammetry (LSV) was conducted using a CHI760E under a constant temperature difference to obtain the current–voltage (*I*–*V*) curves. The scan rate was fixed at 1 mV s^−1^. The output power density (*P*) and energy density (*E*) were calculated according to Equations (4) and (5), respectively:

(4) $$P=\frac{V\cdot I}{S}$$

(5) $$E=\int_{0}^{t}P\left(t\right)dt$$

where *V* and *I* are the output voltage and current at a given moment, respectively, and *S* is the effective contact area between the electrodes and the hydrogel. The energy density (*E*) was obtained by integrating the power density over a discharge period.

The thermal conductivity (*κ*) was measured using a XIATECH TC3000E thermal conductivity analyzer based on the transient hot-wire method. In the ideal model of the transient hot-wire method, the medium is assumed to be infinite, and heat conduction is treated as a one-dimensional transient process in the radial direction. An infinitely long linear heat source is embedded in an infinite and homogeneous medium. Once thermal equilibrium is reached, the linear heat source is suddenly heated by a stepwise constant heat flux, resulting in a temperature rise in both the heat source and the surrounding medium. The *κ* of the medium can then be determined from the temperature rise in the linear heat source. Considering the possible influence of moisture migration during thermal conductivity measurements of wet hydrogels, each test was completed within a short measurement period under the same ambient conditions, and the results are reported as the mean value of three independent measurements, with the standard deviation used as the error bar.

In addition, the power factor (*PF_i_*) and the dimensionless figure of merit (*ZT*), which are key parameters for evaluating the performance of thermoelectric materials, were calculated according to Equations (6) and (7), respectively:

(6) $$PF=S_{i}^{2}\sigma$$

(7) $$ZT=\frac{S_{i}^{2}\sigma T}{\kappa }$$

where *T* is the absolute temperature, and *κ* is the thermal conductivity of the hydrogel.

## 3. Results

In this work, PVA hydrogels were prepared via a freeze–thaw cycling process. During freezing, the crystallization of water molecules compresses the PVA molecular chains, while the abundant hydroxyl groups on the PVA chains interact with one another to form strong hydrogen bonds, thereby constructing a stable network structure. By introducing DMSO as a cosolvent, the hydrogen-bonding interactions between PVA chains can be disrupted because DMSO, with its good solubility and high polarity, acts as a strong hydrogen-bond acceptor. As a result, the aggregation of PVA chains is suppressed, leading to a more open and homogeneous network structure, leading to the formation of a more porous structure within the PVA hydrogel [35]. As shown in Figure 1c, a large number of PVA chains are interconnected within the hydrogel to form a three-dimensional network structure. This structure retains ions and water molecules while providing transport pathways for ion migration.

**Figure 2:**
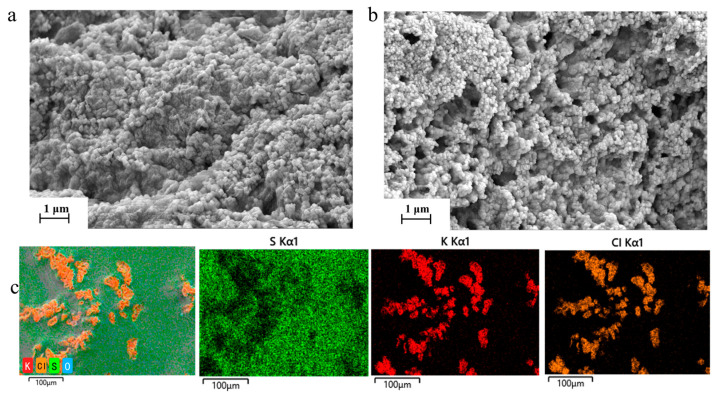
(**a**) Cross-sectional SEM image of the PVA hydrogel without DMSO. (**b**) Cross-sectional SEM image of the PVA-DMSO-KCl hydrogel. (**c**) EDS elemental mapping of the PVA-DMSO-KCl hydrogel.

The influence of DMSO on the hydrogel microstructure was first examined by SEM. As shown in Figure 2a, the PVA hydrogel without DMSO exhibits a relatively compact and less open morphology. In contrast, the PVA-DMSO-KCl hydrogel shows a more porous and interconnected microstructure (Figure 2b). The EDS mapping results further show that S, originating from DMSO, is uniformly distributed throughout the hydrogel, while K and Cl derived from KCl are also well dispersed within the polymer network (Figure 2c). These results indicate that DMSO participates in regulating the polymer-network environment and helps form ion-transport pathways within the hydrogel.

**Figure 3:**
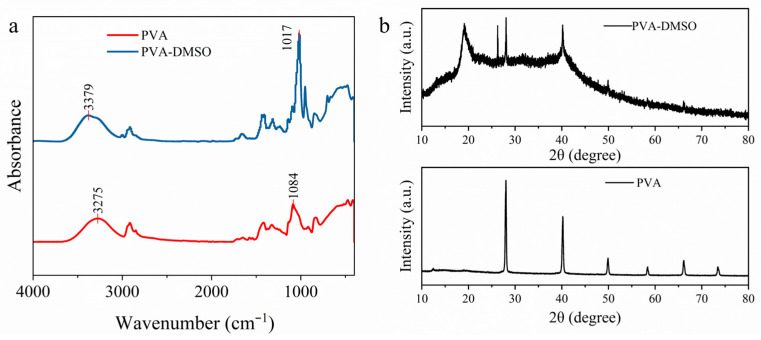
(**a**) FTIR spectra of PVA and PVA-DMSO_30_ hydrogels. (**b**) XRD patterns of PVA and PVA-DMSO_30_ hydrogels.

The FTIR spectra are presented in Figure 3a. The PVA and PVA-DMSO_30_ hydrogels exhibit highly similar overall spectral profiles, indicating that the introduction of DMSO does not induce chemical scission of the PVA main chains or the formation of new covalent bonds. Specifically, pure PVA exhibits a characteristic broad stretching band of hydroxyl groups (–OH) at 3275 cm^−1^, whereas this band shifts markedly to 3379 cm^−1^ in the PVA-DMSO hydrogel. This pronounced shift suggests that the sulfoxide group (S=O) in DMSO, acting as a strong hydrogen-bond acceptor, forms new hydrogen bonds with the hydroxyl groups of PVA, thereby weakening the original hydrogen-bonding network within PVA [36,37,38]. In addition, the PVA-DMSO spectrum shows a pronounced absorption peak at 1017 cm^−1^, which is assigned to the stretching vibration of S=O in DMSO [39]. In the corresponding XRD patterns (Figure 3b), compared with the PVA hydrogel, the PVA-DMSO hydrogel exhibits a broader diffraction peak around 19.1°, while the intensity of the sharp diffraction peaks retained in the PVA sample decreases significantly [40,41]. These changes indicate that the introduction of DMSO markedly reduces the long-range ordering of the PVA chains and increases the fraction of amorphous or short-range ordered regions. This behavior can be attributed to the interaction between DMSO and the hydroxyl groups of PVA, which weakens the original interchain hydrogen bonding and suppresses the regular packing of the PVA chains. The presence of DMSO therefore effectively disrupts the original intermolecular interactions in PVA and induces chain rearrangement. The remaining microcrystalline domains within the gel act as physical crosslinking points, thereby helping maintain the integrity of the hydrogel network. [42].

**Figure 4:**
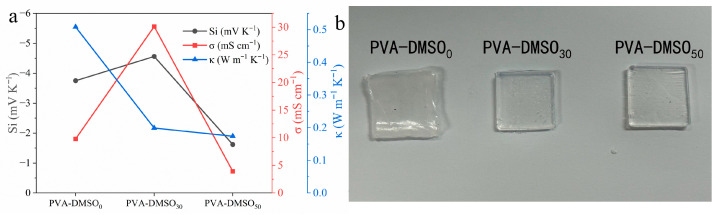
Effect of DMSO content on the thermoelectric and transport properties of PVA-DMSO-KCl hydrogels. (**a**) Ionic Seebeck coefficient, ionic conductivity, and thermal conductivity of PVA-DMSO-KCl hydrogels with different DMSO contents. (**b**) Optical photographs of PVA-DMSO_0_, PVA-DMSO_30_, and PVA-DMSO_50_ hydrogels.

The effect of DMSO content on the thermoelectric and transport properties was further evaluated. As shown in Figure 4a, PVA-DMSO_30_ exhibited the highest *σ* and the largest absolute *S_i_* among the tested compositions, while its *κ* remained at a low level. In contrast, excessive DMSO content in PVA-DMSO_50_ led to a marked decrease in *σ* and *S_i_*, suggesting that too much DMSO may reduce effective ion mobility. The optical photographs in Figure 4b further show that PVA-DMSO_30_ maintained good macroscopic integrity. Based on these results, PVA-DMSO_30_ was selected as the electrolyte for the following electrode-regulated *n*-type thermoelectric device.

**Figure 5:**
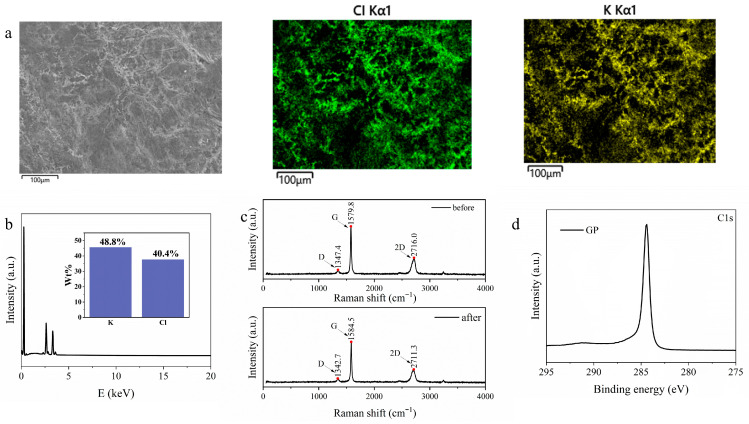
(**a**) SEM image of the surface of the GP electrode after sealed contact with the PVA-DMSO-KCl hydrogel for 24 h, together with the corresponding EDS maps of K and Cl on the electrode surface (**b**) Quantitative comparison of the surface atomic contents of K and Cl detected on the GP electrode after contact with the hydrogel; (**c**) Raman spectra of the GP electrode before and after ion adsorption; (**d**) High-resolution XPS C 1s spectrum of the pristine GP electrode.

To gain deeper insight into the specific interfacial mechanism between the electrode and the hydrogel, a series of experiments was conducted. The GP electrode was attached to the surface of the PVA-DMSO hydrogel and kept sealed in contact for 24 h, after which EDS analysis was performed on the electrode-contacting surface, as shown in Figure 5a. The GP electrode exhibited a relatively rough surface, which provided potential ion-adsorption sites. After prolonged contact with the hydrogel, substantial amounts of K and Cl were detected on the electrode surface. Further quantitative analysis (Figure 5b) revealed that the mass fraction of K on the GP surface was higher than that of Cl. This K/Cl atomic ratio greater than 1 suggests that the GP electrode did not simply adsorb K^+^ and Cl^−^ from the hydrogel in a nonselective manner, but instead exhibited pronounced selective adsorption of K^+^. To further elucidate the adsorption mechanism, Raman spectroscopy was performed on the GP electrodes before and after ion adsorption, and the results are shown in Figure 5c. Prior to ion adsorption, the Raman spectrum of GP exhibits a prominent G band at 1579.8 cm^−1^, a relatively weak D band at 1347.4 cm^−1^, and a well-defined 2D band at 2716 cm^−1^, indicating that GP is a highly graphitized carbon material with long-range structural order, dominant sp^2^-hybridized carbon, and a very low defect density, which is consistent with the typical Raman features of highly graphitized materials [43,44,45]. After ion adsorption, the Raman spectrum of GP shows a blueshift of the G band, together with a blueshift and intensity decrease in the 2D band, while the D/G intensity ratio increases only slightly. These spectral changes suggest that ion adsorption modifies the electronic environment of the GP surface [46,47]. XPS analysis was further carried out on the GP electrodes. As shown in Figure S1, the GP surface contains as much as 97.44% carbon, whereas the oxygen content is only 2.56%. The high-resolution C 1s spectrum (Figure 5d) shows a main peak located at 284.43 eV, further confirming the presence of abundant sp^2^-hybridized C=C bonds on the GP surface. These bonds give rise to a highly conjugated delocalized π-electron-rich structure [48,49]. It is therefore reasonable to infer that the strong adsorption of K^+^ by GP originates from cation–π interactions. This strong noncovalent interaction enables the electron-rich GP surface to selectively interact with positively charged K^+^ and thereby suppress its thermodiffusion [50,51].

**Figure 6:**
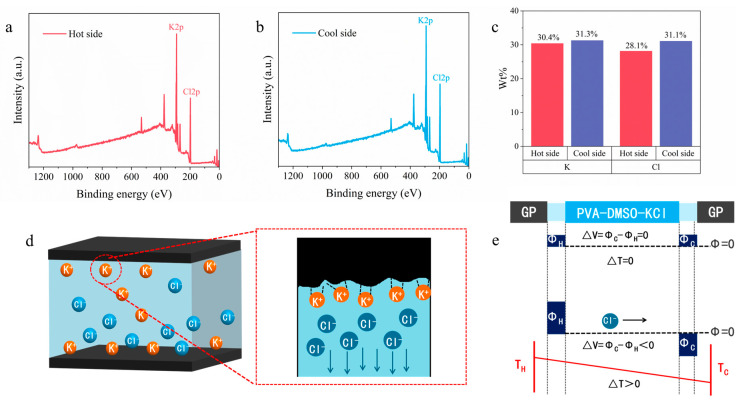
(**a**) XPS spectrum of the hydrogel surface at the hot side; (**b**) XPS spectrum of the hydrogel surface at the cold side; (**c**) comparison of the mass fractions of K and Cl at the hot and cold sides; (**d**) schematic illustration of the microscopic mechanism of selective K^+^ adsorption on the GP electrode surface and anion-dominated thermodiffusion; (**e**) mechanism of potential generation in the GP electrode-based system.

To examine whether this interfacial effect operates in an i-TE device, the PVA-DMSO hydrogel and GP electrodes were assembled into a device (PVA-GP). When the thermovoltage reached a steady state under a temperature difference applied across the two ends of PVA-GP, the hydrogel was rapidly cut from the middle to separate the cold side from the hot side. Subsequently, XPS measurements were performed on the hydrogel surfaces that had been in contact with the electrodes, and the results are shown in Figure 6a,b. Substantial amounts of K and Cl were detected on the hydrogel surface at both the cold side and the hot side. Further quantitative analysis (Figure 6c) showed that, in the PVA-GP system, the concentration difference in K^+^ between the cold and hot sides was only 0.87%, which was much smaller than that of Cl^−^ (2.94%). Because ions tend to migrate toward the lower-energy side under a temperature gradient, the cold side exhibited higher mass fraction of both K and Cl than the hot side. However, due to preferential interfacial adsorption of K^+^ by GP, part of the K^+^ was confined at the electrode/hydrogel interface and could not migrate freely, as shown in Figure 6d. Figure 6e illustrates the mechanism for the generation of negative thermovoltage under a temperature gradient. At room temperature, a large number of cations accumulate at the electrode/electrolyte interface because of cation–π interactions, yet the overall potential difference of the device remains close to zero. Under a temperature gradient, however, a larger fraction of Cl^−^ migrates toward the cold side, while K^+^ is restrained by GP. As a result, the cold side becomes more negatively charged and the hot side becomes relatively more positive, thereby giving rise to a negative thermovoltage in the device. A comparison of the EIS spectra of PVA-GP and PVA-Cu (Figure S2) shows that the two devices exhibit comparable impedance. In the Nyquist plots, PVA-GP and PVA-Cu show similar impedance responses under the same geometric configuration, with no pronounced enlargement of the interfacial impedance for the GP-based device. This indicates that the selective confinement of K^+^ at the GP interface mainly regulates ionic thermodiffusion rather than introducing a substantial interfacial charge-transfer barrier. This feature is crucial for achieving high-performance *n*-type i-TE devices.

**Figure 7:**
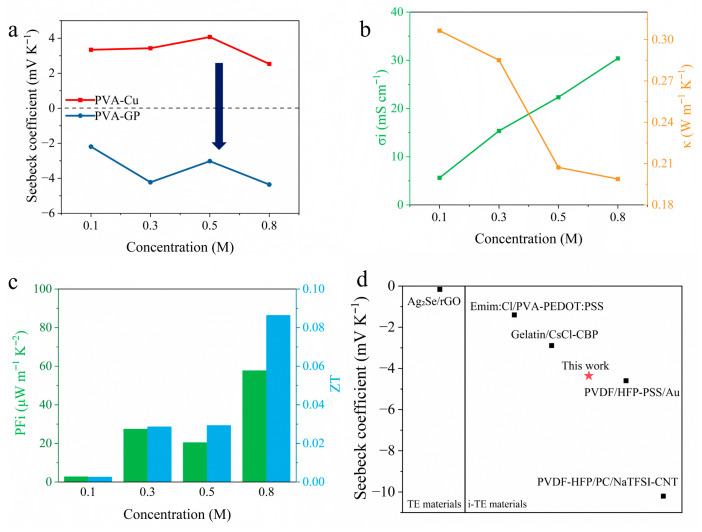
(**a**) Comparison of the *S_i_* of PVA-based i-TE material with conventional Cu electrodes and PVA-GP devices at different KCl concentrations; (**b**) variation in ionic conductivity and *κ* with KCl concentration; (**c**) power factor (*PF_i_*) and figure of merit (*ZT*) at different KCl concentrations; (**d**) comparison of the *S_i_* of this work with those of reported electrode-regulated *n*-type i-TE devices and conventional electronic thermoelectric materials [28,32,34,52,53].

To evaluate the detailed thermoelectric performance of PVA-GP, its properties were first investigated at different KCl concentrations. As shown in Figure 7a, the *S_i_* of PVA-GP remained consistently negative, indicating that Cl^−^ dominated the thermodiffusion process, and its absolute value reached a maximum at 0.8 M (−4.36 ± 0.26 mV K^−1^). Stable *n*-type behavior was maintained over the entire range of ion-donor concentrations (Figure S3). High *σ* and low *κ* are also required for improving the thermoelectric output. The EIS curves at different KCl concentrations were measured using an electrochemical workstation, and the results are shown in Figure S4. Figure 7b presents the variations in *σ* and *κ* of the PVA-DMSO hydrogel as a function of KCl concentration. As expected, the *σ* increased with increasing KCl concentration. Meanwhile, the *κ* of the PVA-DMSO hydrogel remained at a relatively low level throughout the tested range and further decreased as the KCl concentration increased. Based on these parameters, the *PF_i_* and figure of merit (*ZT*) of PVA-GP at different ion-donor concentrations were calculated, as shown in Figure 7c. The maximum *PF_i_* of PVA-GP reached 57.668 μW m^−1^ K^−2^ at a KCl concentration of 0.8 M. In addition, when the KCl concentration was 0.8 M, the *ZT* value of PVA-GP at room temperature (298 K) was 0.0864. The PVA-GP device developed in this work was further compared with recently reported *n*-type i-TE devices based on electrode regulation, as shown in Figure 7d. Its *S_i_* is much higher than that of conventional electronic thermoelectric materials and exceeds those of most reported electrode-regulated *n*-type i-TE devices., approaching the state-of-the-art level in this field. Although it has not yet reached the highest reported value, this system can deliver strong *n*-type i-TE output without relying on highly complicated material design or expensive electrode materials. It therefore exhibits clear advantages in terms of device fabrication simplicity, general applicability, and potential for practical applications.

**Figure 8:**
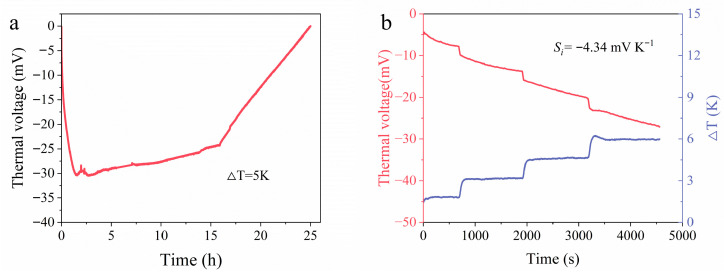
Operational stability and recovery behavior of the encapsulated PVA-GP device. (**a**) Thermovoltage evolution of the simply encapsulated PVA-GP device under a constant temperature difference of 5 K for more than 24 h. (**b**) Thermovoltage response of the device after immersion in KCl solution for 1 min, showing the recovery of the *n*-type *S_i_*.

Operational stability is a critical issue for hydrogel-based i-TE devices because water evaporation can gradually change the ion concentration, internal resistance, and interfacial charge distribution. To further evaluate the operational stability of the device, an extended stability test was conducted using a simply encapsulated PVA-GP device under a constant temperature difference of 5 K. As shown in Figure 8a, the device generated a negative thermovoltage during the initial stage, confirming that the GP-induced *n*-type response was maintained. However, the thermovoltage gradually decayed during prolonged operation and approached zero after more than 24 h. This result indicates that simple encapsulation could delay but not completely suppress water loss and the resulting change in ionic transport.

After the 24 h test, the hydrogel mass decreased from 2.287 g to 1.941 g, and its *σ* decreased from 22.33 to 17.42 mS cm^−1^, corresponding to a conductivity retention of approximately 78.0% (Figure S5). These results confirm that partial dehydration of the hydrogel electrolyte remained the main factor limiting continuous long-term operation. To further examine whether this degradation was irreversible, the tested hydrogel was immersed in KCl solution for 1 min and then reassembled for thermoelectric measurement. As shown in Figure 8b, the recovered device exhibited a linear thermovoltage response with an ionic *S_i_* of −4.34 mV K^−1^, which is very close to the initial value of the optimized PVA-GP device. This result suggests that the loss of thermoelectric output during the 24 h test was mainly associated with reversible water/ion loss rather than irreversible failure of the PVA hydrogel or the electrode-regulated *n*-type mechanism. Therefore, the present PVA-GP device demonstrates recoverable *n*-type thermoelectric behavior, but its continuous long-term operation remains limited by water retention of the hydrogel electrolyte. Further optimization, including more effective encapsulation, anti-drying organohydrogel or ionogel design, and electrolyte-reservoir-assisted device structures, will be necessary for practical continuous energy harvesting.

**Figure 9:**
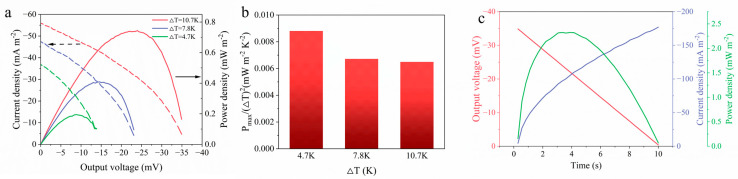
(**a**) Output voltage–current (*I*–*V*) curves and power density curves under different temperature differences; (**b**) variation in the normalized maximum power density with temperature difference; (**c**) evolution of the output voltage and power density of PVA-GP during the discharge process within 10 s.

To evaluate the power-output capability of PVA-GP under practical circuit loads, the *I–V* curves and the corresponding output power density curves at different temperature differences were measured (Figure 9a). The output voltage of PVA-GP decreased with increasing current. Correspondingly, the output power curve exhibited a typical parabolic profile and reached its maximum when the load resistance matched the internal resistance of the device. By varying the temperature difference, it was found that the peak power increased with increasing output voltage, and the maximum power density reached 0.74 mW m^−2^. In contrast, the normalized output power density decreased with increasing temperature difference (Figure 9b) and decreased as the temperature difference increased. This result suggests that the power output of PVA-GP shows diminishing returns at larger temperature differences, with higher normalized power output at lower temperature differences. When PVA-GP was discharged over 10 s, the corresponding power density curve was obtained (Figure 9c), and the peak power density further increased to 2.33 mW m^−2^, indicating that rapid discharge enabled a higher transient power density of PVA-GP. By integrating the power density curve, the energy density of PVA-GP within 10 s was calculated to be approximately 15.82 mJ m^−2^, demonstrating its relatively high energy density. PVA-GP exhibited favorable power and energy outputs, which can mainly be attributed to the interfacial characteristics arising from its microstructure. GP possesses a porous layered structure that can form extensive three-dimensional contact with the hydrogel, greatly increasing the effective electrode/electrolyte contact area. On the one hand, this large contact area significantly reduces the charge-transfer resistance at the interface, allowing the device to deliver higher output current and power density owing to its reduced internal resistance. On the other hand, as evidenced by the CV and GCD curves of PVA-GP (Figure S6), the rough surface of the GP electrode provides abundant ion-adsorption sites and forms electric double-layer capacitance at the interface with PVA, thereby markedly enhancing ion adsorption and storage capability and significantly improving the energy density of the device during transient discharge.

**Figure 10:**
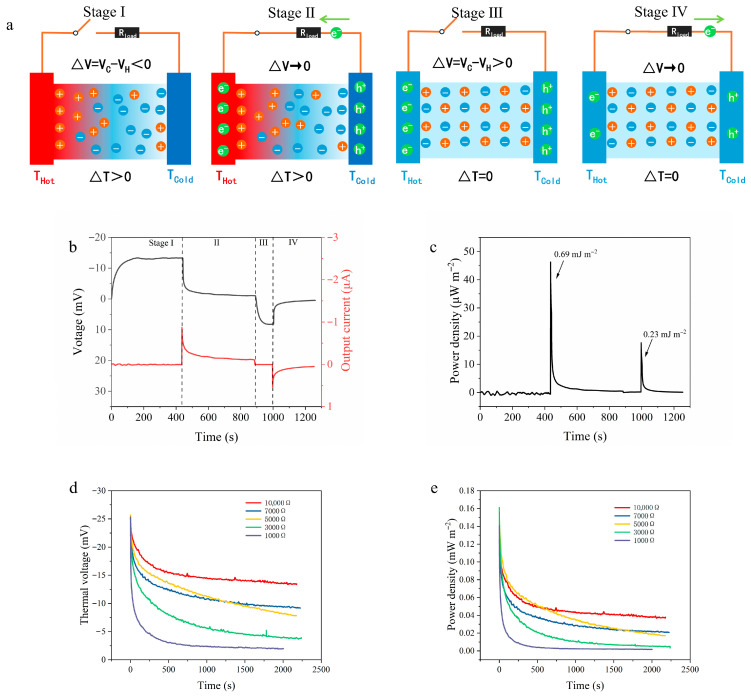
(**a**) Schematic illustration of the four working stages in the charge–discharge cycle of the ITESC; (**b**) evolution curves of the thermovoltage and output current during a complete working cycle; (**c**) comparison of the power density and energy density released in Stage II and Stage IV; (**d**) effect of different external load resistances on the thermovoltage decay process; (**e**) evolution of the output power density over time under different external load resistances.

In addition to directly delivering energy to external circuits, i-TE devices are often employed as energy-storage systems, known as ionic thermoelectric supercapacitors (ITESCs), a concept first proposed by Zhao et al. [54] in 2016. Figure 10a illustrates the complete working process of PVA-GP as an ITESC. The process can be divided into four main stages: (i) A temperature gradient is applied across the device to generate a thermovoltage. (ii) By connecting an external load resistance (*R_load_*), electrons flow from the hot-side electrode to the cold-side electrode to balance the voltage generated by ion migration, during which a rapid voltage drop can be observed. (iii) After disconnecting *R_load_* and removing the temperature gradient, the ionic thermodiffusion disappears and the thermovoltage decays to zero. At this stage, part of the stored charge is dissipated through self-discharge, whereas another part, benefiting from the high specific surface area of the GP electrode, forms an electric double layer with ions in the hydrogel at the electrode/electrolyte interface, causing the open-circuit potential to reverse and become negative. (iv) The ITESC is discharged by connecting it to an external circuit. Figure 10b presents the full voltage evolution of the ITESC during operation. The voltage rapidly decreases when the external circuit is connected, and then a reversed voltage is obtained after the external circuit is disconnected and the temperature gradient is removed. Figure 10c shows the variation in instantaneous power density with time during different stages. The maximum power density in stage II is significantly higher than that in stage IV. This difference arises from the self-discharge behavior of the supercapacitor. After charging, part of the stored energy is dissipated due to leakage current and charge redistribution, resulting in a lower voltage in stage IV than in stage II, which also leads to a reduced energy density in stage IV. The energy densities calculated over a 200 s discharge period are 0.69 mJ m^−2^ for stage II and 0.23 mJ m^−2^ for stage IV.

To further investigate the discharge characteristics of the PVA-GP ITESC under practical power-supply conditions, the energy-release process of the device was evaluated under different external load resistances. Figure 10d shows the decay of thermovoltage with time when PVA-GP was connected to different load resistances ranging from 1000 to 10,000 Ω during long-term discharge. It can be seen that a smaller load resistance, such as 1000 Ω, causes the charges accumulated in the capacitor to be rapidly consumed by the external circuit, resulting in a sharp decrease in thermovoltage. In contrast, a larger load resistance, such as 10,000 Ω, significantly increases the time constant of the circuit and effectively slows down the charge leakage rate, thereby making the thermovoltage decay more gradual and allowing the device to maintain an effective potential difference for a longer period. The corresponding instantaneous power density curves in Figure 10e further confirm this behavior. At the initial stage of discharge, the output power density under each resistance reaches a peak; however, as the discharge proceeds, the high-resistance load (10,000 Ω) exhibits superior power-retention capability and can provide more sustained and stable energy output. This load-dependent discharge behavior indicates that, by rationally matching the impedance of the external circuit, the PVA-GP device can flexibly regulate both the energy-release rate and the power-supply duration, demonstrating considerable practical potential for low-grade heat harvesting and intermittent energy-storage power supply.

## 4. Conclusions

In summary, this work demonstrates a simple electrode-induced strategy for constructing *n*-type i-TE devices by integrating PVA-DMSO-KCl hydrogel with GP electrodes. Unlike many reported *n*-type i-TE systems that rely on specially designed polymer matrices, complex ionic dopants, or expensive electrode materials, the present device realizes polarity conversion from *p*-type to *n*-type by replacing conventional Cu electrodes with GP electrodes. This highlights the important role of the electrode/electrolyte interface in regulating ionic thermodiffusion.

The combined EDS, Raman, XPS, and thermovoltage results suggest that the graphitized, π-electron-rich surface of GP preferentially interacts with K^+^, most plausibly through cation–π interactions. This interfacial interaction is proposed to partially restrain K^+^ thermodiffusion and enables Cl^−^-dominated ion migration under a temperature gradient, thereby generating a stable negative thermovoltage. EIS comparison between PVA-GP and PVA-Cu further shows that the use of GP does not introduce a pronounced increase in internal resistance, indicating that the enhanced *n*-type response is not achieved at the expense of significantly impaired charge transport.

The optimized PVA-GP device delivered a maximum *S_i_* of −4.36 ± 0.26 mV K^−1^, a *PF_i_* of 57.668 μW m^−1^ K^−2^, and a room-temperature *ZT* of 0.0864. Although these values are moderate compared with some state-of-the-art *n*-type i-TE materials, the device exhibits clear advantages in terms of structural simplicity, low-cost electrode selection, and ease of fabrication. In addition, the device achieved a peak transient power density of 2.33 mW m^−2^ during short-time discharge and could operate as an ITESC owing to the porous layered structure and large interfacial area of GP electrodes. These results indicate the potential of GP-based electrode engineering for simultaneous low-grade heat harvesting and transient energy storage. This study provides a feasible and scalable route for designing *n*-type i-TE devices through electrode/electrolyte interfacial regulation.

Nevertheless, the proposed cation–π interaction mechanism is mainly supported by indirect spectroscopic and interfacial evidence at the present stage. In future research, further quantitative investigations on ion adsorption energetics and ion-transport kinetics will be valuable for establishing a more comprehensive mechanistic understanding. In addition, hydrogel dehydration remains a major limitation for long-term operation. Although the n-type Si could be recovered after brief KCl replenishment, further improvements in encapsulation, water retention, and anti-drying electrolyte design are still required to enhance device durability for continuous energy-harvesting applications.

## Data Availability

The original contributions presented in this study are included in the article/Supplementary Materials. Further inquiries can be directed to the corresponding author.

## Supplementary Materials

The following supporting information can be downloaded at: https://www.mdpi.com/article/10.3390/ma19102029/s1, Figure S1. XPS survey spectrum of the pristine GP electrode, showing that carbon is the dominant surface element and confirming the carbon-rich surface nature of the electrode; Figure S2. Comparison of the electrochemical impedance spectroscopy (EIS) spectra of PVA-GP and PVA-Cu devices measured under the same geometric configuration. The similar impedance responses indicate that replacing Cu with GP does not significantly increase the internal resistance of the device; Figure S3. Thermovoltage (Δ*V*) as a function of temperature difference (Δ*T*) for PVA-DMSO-KCl devices with different KCl concentrations using (a) Cu electrodes and (b) GP electrodes. The *S_i_* was determined from the slope of the linear fitting of Δ*V* versus Δ*T* for each sample; Figure S4. EIS spectra of PVA-DMSO-KCl hydrogels with different KCl concentrations measured using stainless-steel electrodes over the frequency range from 10 kHz to 1 Hz. The bulk resistance extracted from the spectra was used to calculate the *σ*; Figure S5. EIS spectra of the PVA-GP device measured after long-term operation; Figure S6. Electrochemical capacitive behavior of the PVA-GP device. (a) Cyclic voltammetry (CV) curves measured at different scan rates. (b) Galvanostatic charge–discharge (GCD) curves measured at 0.1 mA. The results support the interfacial ion-storage capability of the GP-based device.
