# Supplementary material for: An n-Type Ionic Thermoelectric Device Enabled by Synergistic Interactions Between Electrodes and PVA Hydrogel
Source: Materials (Basel). 2026 May 13;19(10):2029. doi: 10.3390/ma19102029 (PMC13208145; doi:10.3390/ma19102029)
Supplement: Supplementary file 1 [file materials-19-02029-s001.zip › materials-4253655-supplementary.pdf]

Supporting information for

An n-Type Ionic Thermoelectric Device Enabled by Synergistic Interactions Between Electrodes and PVA Hydrogels

Changsheng Ye and Xin Shan *

Key Laboratory of Advanced Civil Engineering Materials (Ministry of Education), School of Materials Science and Engineering, Tongji University, Shanghai 201804, China; 2331565@tongji.edu.cn

* Correspondence: shan0055@e.ntu.edu.sg or shanxin@tongji.edu.cn

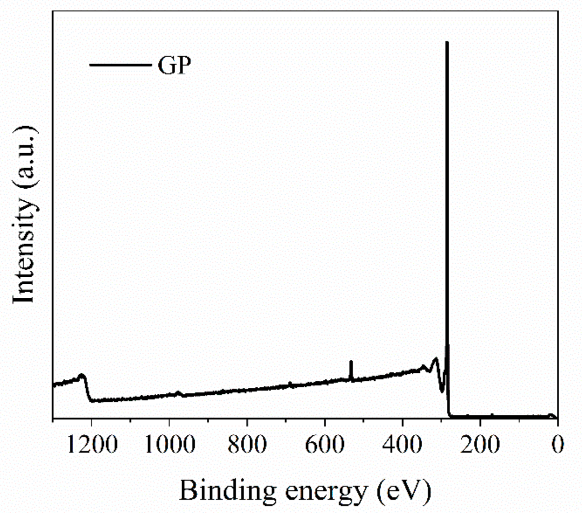

Figure S1. XPS survey spectrum of the pristine GP electrode, showing that carbon is the dominant surface element and confirming the carbon-rich surface nature of the electrode.

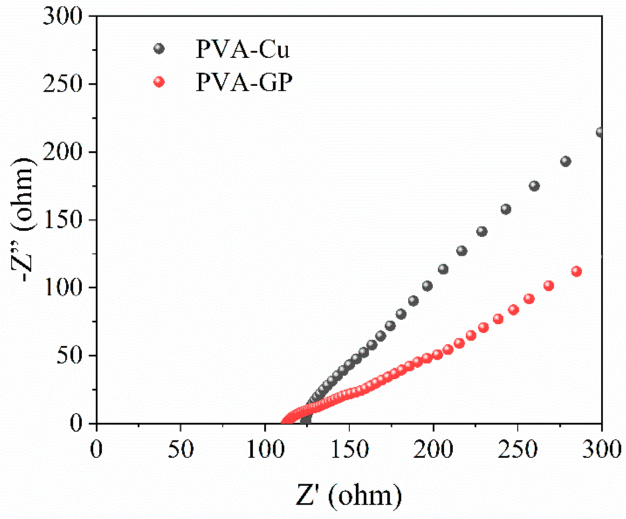

Figure S2. Comparison of the electrochemical impedance spectroscopy (EIS) spectra of PVA-GP and PVA-Cu devices measured under the same geometric configuration. The similar impedance responses indicate that replacing Cu with GP does not significantly increase the internal resistance of the device.

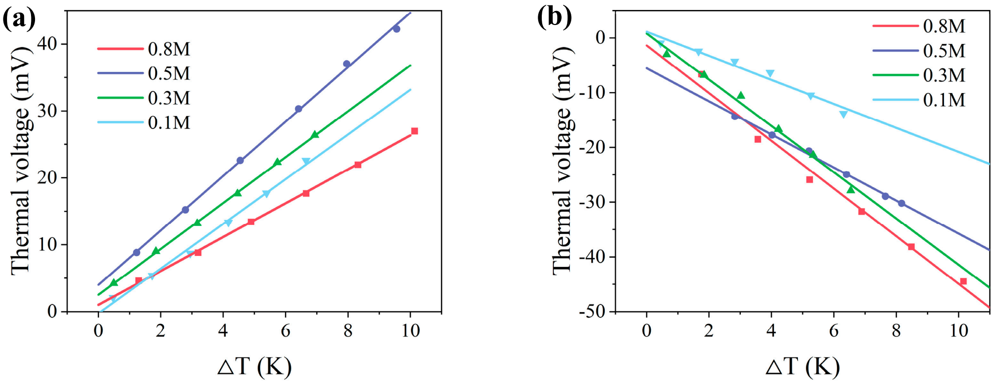

Figure S3.Thermovoltage (ΔV) as a function of temperature difference (ΔT) for PVA-DMSO-KCl devices with different KCl concentrations using (a) Cu electrodes and (b)GP electrodes. The ionic Seebeck coefficient was determined from the slope of the linear fitting of ΔV versus ΔT for each sample.

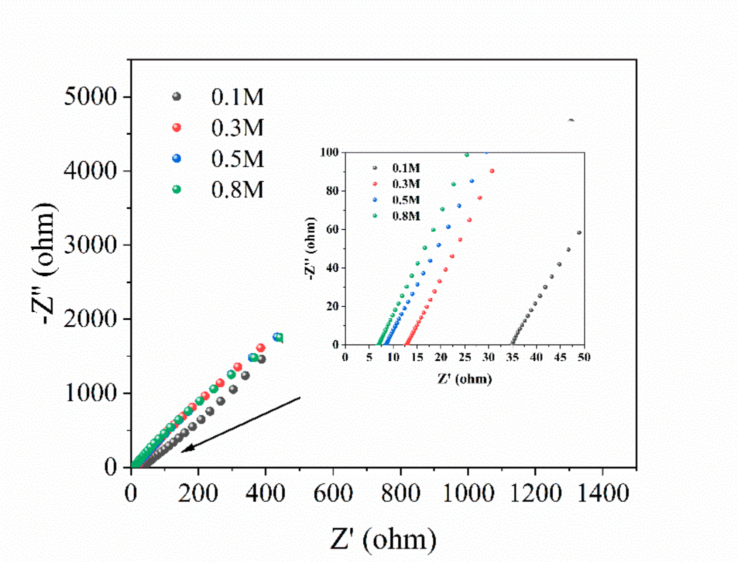

Figure S4. EIS spectra of PVA-DMSO-KCl hydrogels with different KCl concentrations measured using stainless-steel electrodes over the frequency range from 10 kHz to 1 Hz. The bulk resistance extracted from the spectra was used to calculate the ionic conductivity.

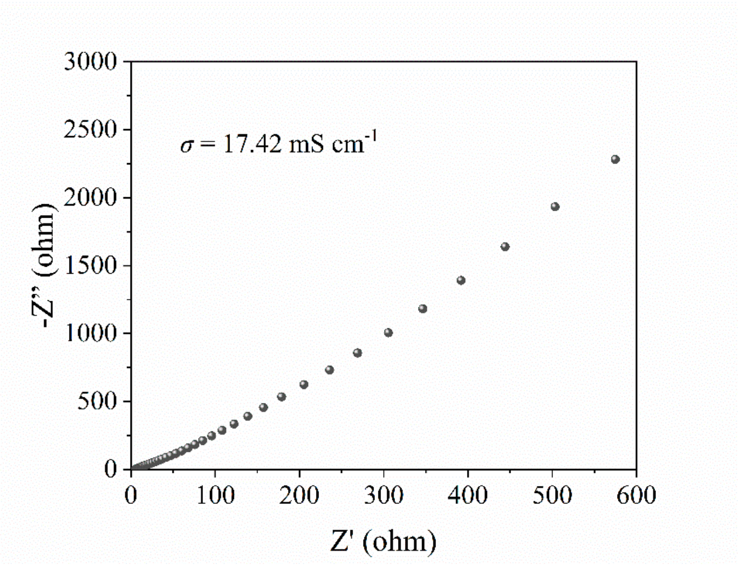

Figure S5. EIS spectra of the PVA-GP device measured after long-term operation

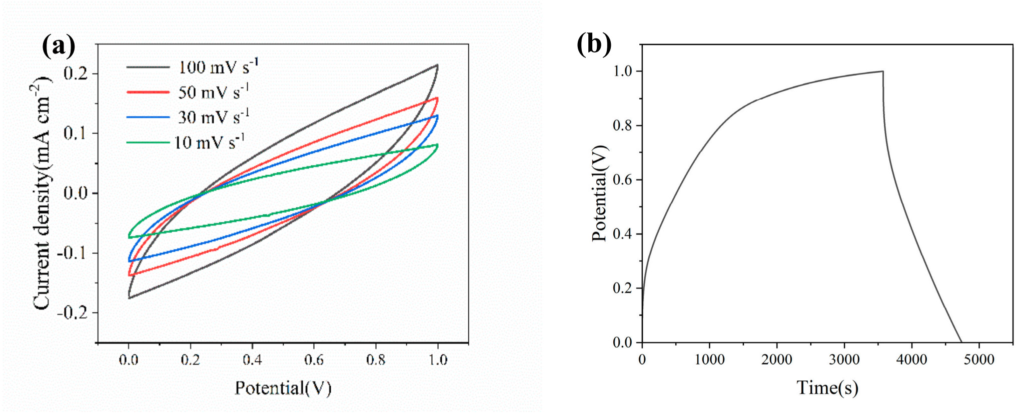

Figure S6. Electrochemical capacitive behavior of the PVA-GP device. (a) Cyclic voltammetry (CV) curves measured at different scan rates. (b) Galvanostatic charge–discharge (GCD) curves measured at 0.1 mA . The results support the interfacial ion-storage capability of the GP-based device
